# The incidence and risk factors for incarceration for violence after traumatic brain injury

**DOI:** 10.1186/s40352-026-00429-w

**Published:** 2026-07-01

**Authors:** Tahnee Guala, Dominique de Andrade, Travis Harries, Amy Langbein, Lorraine Tonner, Harry Hill, Peter Miller, Ashlee Curtis

**Affiliations:** 1 Deakin University, Burwood, Australia; 2 Griffith University, Brisbane, Australia; 3 Barwon Health, Geelong, Australia

**Keywords:** Traumatic brain injury, Incarceration, Violence, Risk factors, Crime prevention

## Abstract

**Background:**

There is increasing evidence to suggest that Traumatic Brain Injury (TBI) is implicated in increased incarceration for violence. TBI is commonly experienced by vulnerable populations who are at greater risk of violent offending due to health and social comorbidities. However, the impact of comorbid risk on outcomes of incarceration for violence is underexplored. Through retrospective data linkage, the current study aimed to identify the rate of incarceration for violence post-TBI and explore the impact of key risk factors in a cohort of 5899 survivors of TBI identified through hospital emergency department (in Victoria, Australia) presentations for TBI (age: *M* = 21.60, *SD* = 2.29; sex: males *n* = 4130, 70.01%, females *n* = 1769, 29.99%; Indigenous status: Indigenous Australian *n* = 136, 2.31%, non-Indigenous Australian *n* = 5763, 97.69%).

**Results:**

The incidence rate for incarceration for violence was 409.31 per 100,000 person-years with incarceration occurring, on average, 2.63 (*IQR* = 4.56) years post-TBI. Risk of incarceration was almost four times higher for individuals who reported pre-TBI incarceration for violence (HR = 3.80, 95% CI [1.89–7.69]) but was not impacted by pre-TBI incarceration for non-violent crimes. Post-TBI incarceration for non-violent crimes demonstrated an increased risk that ranged from 11 (one incarceration, 95% CI [6.66–19.43]) to almost 25 times higher (four or more incarcerations 95% CI [9.42–64.39]) compared to individuals who were not incarcerated. Pre-and-post-TBI substance and substance use treatment demonstrated an increased risk for incarceration for violence that was 17 (pre-TBI: HR = 17.27 95% CI [11.44–26.06]), 19 (post-TBI: HR = 19.32, 95% CI [13.32–28.02]), and almost three (substance use treatment: HR = 2.46–4.67, 95% CI [1.55–18.53]) times higher than those without indicators of substance use or substance use treatment.. Comparatively, only pre-TBI mental health (HR = 4.16, 95% CI [2.62–6.57]) and mental health treatment (HR = 2.68–5.3, 95% CI [1.11–28.03]) significantly increased risk by four and almost three times respectively.

**Conclusions:**

The findings suggest TBI is a key risk factor for incarceration for violence that is exacerbated by health and social vulnerabilities experienced pre-TBI and post-TBI. There may be key opportunities prior to prison contact and after release from prison to provide appropriate intervention that accommodates for TBI-related changes and effectively addresses needs before they manifest into violent offending outcomes.

## Introduction

Violent offending is estimated to occur in 1% of the population (Falk et al., 2014). However, the proportion of individuals of that engage in violent offending is higher for those with traumatic brain injury (TBI). TBI is a form of acquired brain injury that refers to injuries sustained through external causes (Goldman et al., 2022) and it may increase the likelihood of violent offending and contribute to increased concentration of TBI within incarcerated populations (Clasby et al., 2020; Schofield et al., 2015; Williams et al., 2018).For example, Fazel et al., (2011) found that individuals with TBI committed violent crimes at a rate almost three times higher than population controls. Although there is a paucity of research investigating rates of incarceration for violence within general samples (i.e., not exclusively justice-involved)persons with TBI over a longitudinal period, and the impact that contributing factors (e.g., comorbidities such as poor mental health or substance use) may have on such outcomes is underexplored (De Geus et al., 2024). Enhanced understanding of the role of TBI in incarceration for violence can identify critical time points for intervention, and relevant services where risk factors can be targeted to disrupt violent outcomes and reduce the harms associated with violent crime.

The neuropsychological consequences of TBI (e.g., impulsivity, disinhibition, and emotion dysregulation) are known to increase propensity for violence (Clark et al., 2020; Mitchell et al., 2017; Williams et al., 2010). For example, executive dysfunction and social skill challenges can increase violence through greater perception of social input as hostile and behavioural dyscontrol that reduces prosocial decision-making (Clark et al., 2020; Neumann et al., 2017; Schofield et al., 2015). Furthermore, the health and social factors associated with at-risk groups (individuals who have a higher likelihood of experiencing negative outcomes) may be compounded by the neuropsychological impacts of TBI. Factors such as younger age (e.g., 18–25), male sex, and Indigenous status have been associated with increased risk of sustaining a TBI and poor health and social outcomes post-TBI (Clark et al., 2020; De Geus et al., 2024; Fazel et al., 2011; Lakhani et al., 2017; Schofield et al., 2015), but their impact on incarceration for violence is less clear.

Health factors such as poor mental health and risky substance use are associated with violence and offending for individuals with TBI (Jackson et al., 2011; McMillan et al., 2023). Greater frequency or severity of poor health (McKinlay & Albicini, 2016) or difficulties accessing treatment (Nagele et al., 2018; West, 2011) can increase risk of incarceration for violence for persons with TBI. For example, McMillan et al., (2023) found that young male individuals in prison with TBI scored two-and-a-half times higher on a depression measure than male individuals in prisons without TBI. However, it can be difficult to discern whether violence occurs because TBI leads to mental illness or substance use or whether the effects of pre-existing health issues are exacerbated by TBI (Beaulieu-Bonneau et al., 2018; Clark et al., 2020; Horn & Lutz, 2016; Schofield et al., 2015). Indeed, pre-TBI substance use and poor mental health are commonly reported by survivors of TBI who exhibit high levels of aggression and violence in both offending and non-offending samples (McKinlay & Albicini, 2016; Roy et al., 2017), although comparisons with control groups are limited. Further investigation of pre- and -post-TBI health outcomes and the role they may play in incarceration for violence is needed.

TBI may lead to criminal justice contact, but it may also be a consequence of prior contact. Pre-injury behaviour, such as increased propensity to engage in aggressive or confrontational behaviours, can increase the likelihood of sustaining a TBI, but also exacerbate post-injury antisocial tendencies (Clark et al., 2020; Jansen, 2020; Lane et al., 2017). Indeed, Turkstra et al., 2003 posit that pre-injury aggression provides a more accurate indication of post-injury violence than the TBI itself. Previous linked data research has excluded pre-TBI offending from their analyses to investigate the causal role of TBI in offending (Schofield et al., 2015). However, prior offending may be associated with ongoing offending (Mulder et al., 2011; Tateno et al., 2003). Thus, exploration of pre-and-post TBI trajectories of offending is imperative to increase understanding of how TBI interacts with social factors in relation to incarceration for violence.

Survivors of TBI often experience high rates of adverse health and social outcomes irrespective of violent offending and both prior to, and after, TBI. Obtaining and retaining large sample sizes of individuals with TBI is difficult, resulting in many studies having low sample sizes, limited generalisability (Zarzycki et al., 2024), and therefore an inability to draw meaningful conclusions about how TBI may impact violent offending and the influence of confounding factors on this relationship. In particular, it is challenging to ascertain the role of such factors without a sample of individuals with TBI who engage in violent offending to individuals with TBI who do not offend. Moreover, the potential impact of pre-TBI health and social risk factors (e.g., poor mental health and offending) is often not accounted for or is not accurately accounted for (e.g., due to the limitations of self-report data). However, it is important to understand whether incarceration for violence after TBI is associated with the exacerbation of baseline vulnerabilities, or the onset of new vulnerabilities, or a combination of both. This knowledge can inform the utilisation of screening tools based pertinent risk factors, in addition to identifying when interventions are needed and what interventions are needed based on specific risk factors. Linked data methodologies provide a promising avenue for more accurate research as it mitigates many of the limitations associated with other methods (e.g., active participation is not required, and large sample sizes can be obtained; Boyd et al., 2012) and it can permit identification of specific timepoints where intervention is required to disrupt violent offending.

The influence of contributing factors warrants further exploration as improved knowledge of their impacts can inform the development of targeted intervention for key needs that are delivered to at-risk groups (De Geus et al., 2024). It is challenging to investigate the role of TBI in incarceration for violence and the impact of multiple contributing factors whilst accounting for pre-TBI vulnerabilities and demographic confounders (e.g., sex, Indigenous status, age). Research utilising population-based methods such as data linkage are beneficial for understanding how pre-and-post TBI factors impact risk of incarceration related to violence while controlling for various confounding factors. Thus, the aim of this study is first, to examine post-TBI incarceration for violence through retrospective data linkage of a population-based cohort of young adults who sustain a TBI when aged 18–25 (identified through presentation to hospital emergency departments). Second, to investigate the impact of pre-and-post-TBI contributing factors (incarceration history, mental health, and substance use) on the relationship between TBI and incarceration for violence. This study is guided by the following research question:

1. What is the rate of incarceration for violent offending in young adults with TBI?
2. How is risk of incarceration for violence impacted by health (mental health and substance use) and social outcomes (prior offending)?

## Method

Ethics approval was obtained from the Justice Human Research Ethics Committee (CF/23/8005), *REDACTED FOR PEER REVIEW* (2023–235), and *REDACTED FOR PEER REVIEW* (2025/190). Retrospective data linkage was employed through the Centre for Victorian Data Linkage (CVDL) to construct a chronological sequence of events from the timepoint of the index TBI to index incarceration for violence.

## Cohort

There were 5899 individuals who presented to a public emergency department (ED) in Victoria, Australia for an index TBI (TBI determined using ICD10 codes: S06, S09 S09.7, S09.8 World Health Organisation, 2019) during the 1 st of January 2009- 31 st December 2018, when aged 18–25 (Victorian Emergency Department Minimum Data; VEMD). Individuals with any prior TBI recorded in the ED data (*n* = 20) were excluded to control for the impact for pre-existing injuries. Records were linked to incarceration data (Corrections Victoria; CV), substance use data (Alcohol and Drug Information System and Victorian Alcohol and Drug Collection; ADIS.VADC), mental health data (Clinical Public Mental Health Services; CPMHS), and death data (Victorian Death Index; VDI). The records utilised spanned from the 1 st of January 2001—31st of December 2021. Time at risk ranged from 3–12 years as individuals sustained a TBI at various time-points and entered the study at different times.

## Measures

### Outcome

Index incarceration for violence was determined by the first admission to prison (CV) post-TBI for violent offences/charges per the 2011 Australian and New Zealand Standard Offence Classification system (ANSZOC; Australian Bureau of Statistics, 2011). The offences/charges cover homicide and related offences, acts intended to cause injury, abduction harassment and other offences against the person, and aggravated robbery. Specific details of violent offence/charge classifications^1^ are presented in Appendix Table 1. The incarceration data included periods of remand and sentenced periods, and as such incarceration events for violence were excluded if they occurred less than two months post-TBI to reduce the potential of capturing of offending occurring prior to TBI (*n* = 41).

### Covariates

Covariates were selected a priori based on established literature (e.g., Clark et al., 2020; McKinlay & Albicini, 2016; Schofield et al., 2015) and the availability of administrative data.

#### Demographic covariates

A categorical variable was developed to represent estimated age (18–21; 22–25). Estimated age at TBI was calculated by subtracting the index TBI date from masked birthdate variables (VEMD). Indigenous status was determined by variables that provided Indigenous identification (Aboriginal and Torres Strait Islander, Aboriginal but not Torres Strait Islander, Torres Strait Islander but not Aboriginal, Non-Indigenous, all sources). Sex was identified using variables that indicated male or female sex (Female, male; all sources).

#### Prior incarceration

Two binary flags were created to indicate the presence of incarceration events occurring pre-TBI (CV). The first flag indicated whether any participants had a pre-TBI incarceration for violence (from the full cohort) and the second flag indicated any participants (from the full cohort) that had a pre-TBI incarceration for non-violent offences/charges (any offences/charges exclusive of those considered violent per ANSZOC criteria). For those who were imprisoned post-TBI for non-violent offences, a cumulative time-varying variable was developed to categorise the number of post-TBI incarceration events for the non-violent offence(s) per individual prior to post-TBI index incarceration for violence or study end date (CV). A binary categorical time-varying covariate was created to flag intervals where individual risk of incarceration for violence changed due to being in prison for a prior offence.^2^

#### Risky substance use

Two binary variables were created to represent pre-TBI and post-TBI risky substance use. These variables were created based on data indicating acute substance-related ED presentations (ICD10 codes F10-F19, VEMD) and/or presentations to substance use services (e.g., assessment; ADIS/VADC). A third time-varying cumulative categorical variable was developed to denote the number of post-TBI substance use treatment programs (i.e., participation in an intervention program with presentations for assessment only excluded).

#### Poor mental health

Pre-TBI and post-TBI poor mental health were denoted using two binary variables. These variables were developed using data from flag ED presentations (e.g., ICD10 codes F30-48, F99 VEMD) for acute mental health events and/or presentations to mental health services (e.g., assessment; CPMHS). A third time-varying categorical variable was developed to indicate the cumulative number of mental health treatments experienced post-TBI.

#### COVID-19 restrictions

A binary indicator was used to control for changes in risk from 31 st March 2020-23rd October 2021 when Victoria, Australia was subject to COVID-19 restrictions (e.g., lockdowns; Macreadie, 2022).

### Analysis

Single time-point survival analysis was employed in Stata (Version 18) to model time to incarceration for violence post-TBI (i.e. the failure event). Cox proportional hazards models were computed to evaluate the impact of covariates and time-varying covariates on incarceration for violence. Individuals were no longer considered “at risk” (i.e. right censored) on the 31 st of December 2021 unless they were incarcerated for violence (i.e. failed) or died during time at risk. Separate models for prior incarceration, and substance, and mental health were developed to permit exploratory analysis of these factors. A combined model was not feasible as there were insufficient outcome events to support the number of covariates required, increasing the risk of model overfitting or unstable estimates. Unadjusted cox proportional hazard regression models were run for each covariate followed by the three adjusted models that included selected covariates. Each adjusted model included sociodemographic covariates (age at injury, sex, and Indigenous status) and COVID-19 restrictions (to control for changes in risk during the period in which restrictions were in place). The substance use and mental health models both included an additional covariate ‘incarceration period’ to control for when individuals were in prison for non-violent offences. For each adjusted model, a series of steps were undertaken to test assumptions and measure model fit. Variance Inflation Factors (VIF) was used to detect multicollinearity with variables excluded if VIF exceeded the acceptable threshold (VIF > 5; Kyriazos & Poga, 2023). Model calculation used robust standard errors to account for intragroup correlation and reduce the likelihood of Type 1 errors. The models were built using stepwise selection via Bayesian Information Criterion (BIC) with covariates excluded if they strongly decreased model fit (BIC increase > 10). Schoenfield residuals were examined to test the proportional hazard assumption and covariates that violated assumptions (Schoenfield residuals *p* <.05) were stratified. Complete case analysis was performed as the proportion of individuals with missing data was small (n < = 5%) and thus unlikely to bias results (Mukherjee et al., 2023).

## Results

There was 45,931.014 [95% CI: 0.004–0.005] person-years of data (*Mdn* = 7.06 [*IQR* = 4.663] for the 5899 individuals in the cohort. The average age at injury (TBI) was 21.601 (SD: 2.287) The sample was predominantly male, and the majority of individuals were non-Indigenous. The records indicated a small proportion experienced prior incarceration, risky substance use, poor mental health, and substance use treatments, or mental health treatments (see Table 1).

**Table 1:** Descriptive statistics

Characteristic	n (%)
Sex	
Male	4130 (70.01%)
Female	1769 (29.99%)
Indigenous Status	
Indigenous	136 (2.31%)
Non-Indigenous	5763 (97.69%)
Pre-TBI Incarceration	
Incarceration for Violent Offence	50(0.85%)
Incarceration for Non-violent Offence	29(0.49%)
*Prior Incarceration Number*	
1	115 (1.95%)
2–3	65 (1.1%)
4 +	19 (0.32%)
Risky Substance Use	849 (14.39%)
Pre-TBI	342 (5.8%)
Post-TBI	507 (8.58%)
Substance Treatment	
1	237 (4.02%)
2–3	64 (1.08%)
4 +	10 (0.17%)
Poor Mental Health	687 (11.65%)
Pre-TBI	420 (7.12%)
Post-TBI	267 (4.53%)
Mental Health Treatment	
1	214 (3.63%)
2–3	95 (1.61%)
4 +	27 (0.46%)

The pre-TBI and post-TBI proportions total the number of risky substance use and poor mental health overall. The total is not indicative of individuals exclusively engaging in either pre-TBI or post-TBI risk substance use, and the data is inclusive of individuals of individuals who reported both

### Rate of incarceration for violence

There were 188 (3.19%) individuals who were incarcerated for a violence offence post-TBI, with an incidence rate of 409.31 per 100,000 person-years. For those who were incarcerated for violence, the median time to incarceration was 2.63 years (*IQR* = 4.56). Aggregated time at risk and incidence rates for each covariate is presented in Table 2.

**Table 2:** Time at risk and incidence rates per 100,000 person-years by binary and categorical covariates

Characteristics	Time at Risk	Incidence Rate per 100,000 Person-years
Age at Injury		
18–21	31,464.55	375.03
22–25	14,497.93	482.83
Sex		
Male	32,376.83	521.98
Female	13,585.64	139.85
Indigenous Status		
Indigenous	934.37	2361.55
Non-Indigenous	45,028.1	36.44
Pre-TBI Incarceration		
Incarceration for Violent Offence	158.042	18,982.24
Incarceration for Non-violent Offence	114.67	10,464.62
*Post-TBI Incarceration Category*		
1	547.35	6394.56
2–3	235.726	9757.08
4 +	48.613	20,570.51
Risky Substance Use		
Pre-TBI	2139.69	2336.79
Post-TBI	3946.57	2255.12
Substance Use Treatment		
1	1254.289	2072.89
2–3	223.97	1339.48
4 +	27.614	7242.71
Poor Mental Health		
Pre-TBI	29,892.59	1555.57
Post-TBI	2333.48	385.69
Mental Health Treatment		
1	1121.71	980.65
2–3	424.59	1648.63
4 +	99.21	2015.84
Incarceration Period		
No	45,644.44	411.88
Yes	318.03	0
COVID-19		
No	459,296.44	386.34
Yes	666.04	1951.85

Time at risk refers to the duration in which a participant is actively exposed to the specific event (incarceration for violence); Incidence rate per 100,000 person-years refers to the rate of new events that would occur if 100,000 people were followed for one year

### Cox proportional hazards models

Each covariate was initially examined in a separate model to estimate its unadjusted hazard ratio (see Table 3). Age at injury was removed from all adjusted models at it decreased model fit (BIC > 10) in each model.

**Table 3:** Unadjusted hazard ratios

Covariates	Unadjusted Model	
	**HR**	**95%CI**
Age at Injury		
18–21	1.029	[0.756–1.401]
22–25	0.972	[0.714–1.322]
Sex		
Male	3.753***	[2.335–6.031]
Female	0.266***	[0.166–0.428]
Indigenous Status		
Non-Indigenous	0.152***	[0.091–0.233]
Indigenous	6.578***	[4.286–10.095]
Pre-TBI Incarceration		
*Incarceration for Violent* Offence	50.503***	[32.621–78.189]
*Incarceration for Non-violent* Offence	24.328***	
Post-TBI Incarceration Category		
1	22.902***	[15.733–33.337]
2–3	36.725***	[23.219–58.087]
4 +	74.731***	[38.159–146.353]
Risky Substance Use		
Pre-TBI	7.204***	[5.206–9.968]
Post-TBI	9.607***	[7.246–12.744]
Substance Use Treatment		
1	5.901***	[3.892–8.947]
2–3	3.767*	[1.176–12.059]
4 +	24.908***	[6.143–100.984]
Poor Mental Health		
*Pre-TBI*	4.582***	[3.276–6.409]
*Post-TBI*	0.961	[0.496–1.86]
Mental Health Treatment		
1	2.498**	[1.363–4.578]
2–3	4.781***	[2.288–9.991]
4 +	6.528**	[1.715–24.842]

Significant values are bolded

^*^Significant at <.05, ^**^Significant at <.01, ^***^Significant at <.001

#### Prior incarceration

Table 4 presents the unadjusted and confounder-adjusted model for prior incarceration. No issues with multicollinearity were detected (VIF > 5), and the model was stratified by Indigenous status (Schoenfield residuals *p* = 0.007). The confounder-adjusted model demonstrates that pre-TBI incarceration for violence, post-TBI incarceration for non-violent offences, and the COVID-19 restrictions were associated with increased hazard of incarceration for violence. There was no significant effect of pre-TBI incarceration for non-violent offences. The hazard for incarceration for violence increased with each additional post-TBI incarceration for a non-violent offence. Females had a reduced risk of experiencing incarceration for violence.

**Table 4:** Confounder-adjusted model for prior incarceration

Covariates	HR	95%CI
Age at Injury		
18–21		
22–25		
Sex		
Male		
Female	0.338***	[0.21–5.44]
Indigenous Status		
Non-Indigenous		
Indigenous		
Pre-TBI Incarceration		
*Incarceration for Violent* Offence	3.808***	[1.886–7.69]
*Incarceration for Non-violent* Offence	1.97	[0.868–4.468]
Post-TBI Incarceration Category		
1	11.373***	[6.657–19.43]
2–3	12.375***	[5.811–26.35]
4 +	24.624***	[9.417–64.392]
COVID-19	1***	[1–1.001]

Significant values are bolded

^***^Significant at <.001, Age at Injury removed from the model as it decreased fit; Indigenous Status was stratified in the final model. Estimates for males are not reported in the final model as males were treated as the reference group

#### Substance use

Table 5 presents the confounder-adjusted model for substance use. There were no issues with multicollinearity (VIF < 5) or assumptions. Pre-and-post TBI risky substance use, substance treatment, identifying as Indigenous, and the COVID-19 restrictions significantly increased the hazard of experiencing incarceration for violence. The effect of substance use treatment varied across levels, with 2–3 substance use treatments producing a non-significant effect. The risk of incarceration for violence was decreased for females and during periods of incarceration for non-violent offences.

**Table 5:** Confounder-adjusted model for substance use

Covariates	HR	95%CI
Age at Injury		
18–21		
22–25		
Sex		
Male		
Female	0.252***	[0.156–0.407]
Indigenous Status		
Non-Indigenous		
Indigenous	3.196***	[2.078–4.914]
Risky Substance Use		
Pre-TBI	17.266***	[11.44–26.058]
Post-TBI	19.316***	[13.315–28.02]
Substance Use Treatment		
1	2.463***	[1.546–3.923]
2–3	1.026	[0.282–3.735]
4 +	4.667*	[1.175–18.533]
Incarceration Period	0.599	[0.58–0.619]
COVID-19	1**	[1–1.001]

Significant values are bolded

^*^Significant at <.05, ^**^Significant at <.01, ^***^Significant at <.001 Age at Injury removed from the model as it decreased fit; Estimates for one level of each binary categorical variables, Indigenous status and sex, are omitted in the final model as non-Indigenous Australians and males served as reference groups respectively

#### Mental health

Table 6 presents the unadjusted and confounder-adjusted models for mental health. Multicollinearity was not detected (VIF < 5) and assumption testing led to stratification by Indigenous status (Schoenfield residuals *p* = 0.022). Pre-TBI poor mental health, post-TBI mental health treatment, and the COVID-19 restrictions significantly increased the hazard of incarceration for violence, with the hazard for mental health treatment increasing alongside the number of treatments. Female sex and periods of incarceration for non-violent offences were associated with decreased risk whilst post-TBI poor mental health had no significant impact.

**Table 6:** Confounder-adjusted model for mental health

Covariates	HR	95%CI
Age at Injury		
18–21		
22–25		
Sex		
Male		
Female	0.214***	[0.134–0.323]
Indigenous Status		
Non-Indigenous		
Indigenous		
Poor Mental Health		
*Pre-TBI*	4.145***	[2.617–6.565]
*Post-TBI*	1.03	[0.492–2.159]
Mental Health Treatment		
1	2.678*	[1.11–6.469]
2–3	5.605***	[2.513]
4 +	5.306*	[1.005–28.028]
Incarceration Period	0.575***	[0.557–0.595]
COVID-19 period	1.001**	[1–1.001]

Significant values are bolded

^*^Significant at <.05, ^**^Significant at <.01, ^***^Significant at <.001. Estimates for males are not reported in the final model as males were treated as the reference group

## Discussion

This study aimed to identify the incidence and timing of incarceration for violence post-TBI and investigate the impact of contributing factors. The rate of incarceration for violence reported for young adults with TBI is three times higher than the average rate of incarceration for all types of offending (including non-violent) across all ages in Victoria for the same incarceration period (2009–2021, Sentencing Advisory Council, 2025a, 2025b). Similarly, 3.19% of the sample had been incarcerated for violence which is around three times higher compared to prior literature on the rate of violent crime in the general population (1%; Falk et al., 2014). For young adults with TBI, factors such as multiple pre-and-post-injury incarceration events, worse pre-TBI mental health, and increased pre- and- post-TBI risky substance use contribute to a greater risk of engaging in violent offending.

The current study found that history of incarceration for violent offences pre-TBI and non-violent offences post-TBI is associated with increased risk of incarceration for violent offending after TBI. In line with prior findings, violence pre-injury demonstrated an almost four-fold increase in risk suggesting that prior violent offending may be associated with post-TBI violent offending (Jansen, 2020; Lane et al., 2017; Turkstra et al., 2003). This may be indicative of pre-TBI personality and/or behavioural traits associated with violence such as high levels of hostility (Kerr et al., 2011; Matei et al., 2022). However, the level of risk associated with post-TBI incarceration for non-violent offences was 267%−700% higher than that of pre-TBI violent offending. Individuals who were incarcerated for non-violent offences post-TBI had an 11–24 times greater risk than those who were not. The finding suggests that criminal legal contact more generally is associated with increased likelihood of incarceration for violence for individuals with TBI (Clark et al., 2020; Jansen, 2020; Mulder et al., 2011). A history of offending is also a risk factor for re-incarceration in non-TBI populations (e.g., 39% of people released from Victorian prisons return to prison within two years, Sentencing Advisory Council). Future studies should compare how criminal history impacts later incarceration for violence across individuals with TBI to individuals without TBI to further examine potential differences in risk. It should be noted that while the cohort consisted of individuals presenting to an ED for an index TBI, there may be potential impacts of pre-existing TBIs that were sustained but not captured in ED records (e.g., an individual may not have presented to an ED). Indeed, young adults may be at an increased risk of sustaining repeated TBIs (McKinlay et al., 2008). Moreover, multiple TBIs can increase the severity of neuropsychological impairment and in turn, elevate risk of violence (Horn & Lutz, 2016; Nagele et al., 2018). Future research should employ triangulation of linked data with self-reported TBI data to control for undocumented TBI. Notwithstanding this potential limitation, the study provides evidence that young adults with TBI face marked vulnerability to repeat offending and cyclical contact within the criminal legal system with incarceration for violence a potential consequence of this cycle. As young adults are likely to be at an increased risk of incarceration (Sweeten et al., 2013), further research into incarceration for violence directly comparing young adults with TBI to young adults without TBI is needed.

The current study found a substantially elevated risk of incarceration for violence for survivors of TBI who experience substance use issues, with post-TBI substance use problems (19-fold risk increase) demonstrating a 12% higher risk than pre-TBI substance use (17-fold risk increase). This aligns with prior research suggesting that TBI exerts an exacerbating influence on pre-existing substance use issues (Clark et al., 2020; McKinlay & Albicini, 2016; Schofield et al., 2015) and can lead to increased rates of substance use for individuals who do not have pre-existing substance use issues (e.g., as a coping mechanism; Baguley et al., 2006). Substance use is an independent predictor of violence (Curtis et al., 2021) that may compound neuropsychological challenges by increasing impulsivity and reducing decision-making capacity that in turn, leads to greater frequency or intensity of violence (Grant & Chamberlain, 2014). As substance use has been demonstrated to increase risk of incarceration for violence for survivors of TBI, substance use services may be well positioned to support efforts to mitigate violence by reducing the associated harms of substance use for individuals with TBI.

Mental health issues experienced pre-TBI contributed to a four-fold increased risk of incarceration for violence whereas post-TBI mental health issues did not demonstrate a significant impact. The finding that post-TBI mental health does not play a major role in risk of incarceration for violence is contrary to prior research (Baguley et al., 2006; McKinlay & Albicini, 2016). The current results suggest that, after accounting for both pre- and—post-TBI mental health, pre-existing poor mental health is more salient factor for increased risk of violence. Poor mental health pre-TBI may be associated with greater risk of incarceration for violence due to the compounding impact of neuropsychological changes on emotional dysregulation and emotion recognition (Neumann et al., 2017; Weis et al., 2022). A TBI can lead to decreased understanding of emotions which can increase risk for aggression by exacerbating the negative impacts of pre-existing poor mental health or decreasing adaptive coping strategies (e.g., due to poor emotional insight and increased emotional dysregulation; Jansen, 2020; Neumann et al., 2017). The current findings provide a strong rationale for mental health screening alongside the occurrence of TBI to disrupt violent offending outcomes through early mental health intervention that reduces the impact of unmet mental health needs manifesting as incarceration for violence. However, the potential ineffectiveness of current interventions must also be considered.

The present study found greater risk associated with multiple substance and mental health treatment episodes, indicating an increased severity of these issues that contributes to higher risk of violent offending. Indeed, while one mental health treatment demonstrated an almost three-fold increase, the categories of two–three treatments and four or more treatments exhibited around an almost six-fold increase in risk. Notably, risk of incarceration for violence increased across all levels of substance use treatment (one treatment: almost a three-fold increase; four or more treatments: an almost five-fold increase) except for individuals who underwent 2–3 treatment episodes. The severity of substance use may still play a key role, with more severe issues requiring a greater number of treatment episodes (e.g., recreational use compared to substance dependence). In comparison, the risk associated with a singular treatment episode could be due to differences in treatment completion. Potentially, at-risk individuals may not be effectively engaged in, or complete treatment (Brett et al., 2017) and therefore their needs remain unmet (unaddressed) and contribute to risk of incarceration for violence. Disengagement with treatment is often attributed to low motivation, however low motivation may actually be a result of survivors of TBI encountering ineffective treatment where their needs are not accurately understood or responded to (Guala et al., 2026). This could suggest a need for practitioners to be aware of additional challenges in intervention delivery that may impact motivation for individuals with TBI. Generally, treatment completion is a critical factor for preventing recidivism (Henwood et al., 2015). Further research is needed to understand how substance use and mental health treatment completion may impact incarceration for violence in individuals with TBI.

Comparatively, the current finding regarding the higher risk associated with substance use and mental health service treatment episodes may support emerging discourse suggesting that health treatment may be less effective for individuals with TBI (Norman et al., 2023; Saunders et al., 2025). Services may not routinely screen for TBI resulting in TBI being less likely to be recognised and subsequent treatment may not accommodate for neuropsychological impacts and thus be less effective (Admire & Mitchell, 2010; Horn & Lutz, 2016). For example, survivors of TBI require modified interventions that accommodate for cognitive impairment (e.g., modified materials, greater repetition of concepts, or information handouts to aid retention) but there are few effective TBI-specific interventions for mental health or substance use (Bogner & Corrigan, 2013; Gertler et al., 2015). Moreover, while modified interventions are available in Victorian prisons there are extensive waitlists that limit access to support while incarcerated (Greaves, 2023). Survivors of TBI may also encounter difficulty accessing mental health and substance use treatment after release from prison due to issues with service availability (Lansdell et al., 2018). Additionally, the screening tools and interventions for mental health and substance use demonstrate poor reliability for detecting and responding to the presence of mental health issues in survivors of TBI (e.g., due to lack of modification for cognitive difficulties; Bogner & Corrigan, 2013; Gertler et al., 2015; Rose et al., 2023; Stalder-Lüthy et al., 2013). The risk of violent offending could be reduced by improving identification of TBI through routine screening of TBI in health services, and the development of effective evidence-based interventions that are suitable for individuals with TBI.

The findings of the current study are limited by a number of factors. Our measure of violent offending did not capture police arrests and undetected violent behaviour. This may impact the reliability of the estimations for the frequency timing of violence. Moreover, this may have resulted in higher estimations of incarceration for violence in Indigenous Australian participants as this group is over-represented in prison (Australian Institute of Health & Welfare, 2024). Future research should incorporate data from all stages of the criminal legal system including arrests, sentencing, incarceration, and parole, to obtain an in-depth outline of violent offending trajectories. Additionally, the current study may be limited by an absence of a control group without TBI. Whilst comparison to the general population research suggests vulnerability to incarceration for violence for persons with TBI, direct comparison to individuals without TBI (e.g., population or matched sibling controls) would enhance knowledge. Additionally, females were under-represented in the sample which may reduce the generalisability of the findings. Females are more likely to sustain a TBI through domestic violence victimisation which is less likely to be captured in ED administrative data used in this study. However, TBIs sustained through domestic violence victimisation are implicated in violent offending perpetration in females (O'Sullivan et al., 2015). Thus, the role of sex in the relationship between TBI and violence requires further research particularly as females more often report a TBI prior to their first offence when compared to males (Colantonio et al., 2014; Ferguson et al., 2012). Stratified or quota sampling methods and triangulation with self-report data could be utilised in additional research to obtain more balanced samples when investigating the role of sex in incarceration for violence post-TBI. Lastly, similar to prior linked data TBI research, there were low numbers of participants who experienced post-TBI incarceration, substance use treatment, and mental health treatment, which may have contributed to wider confidence intervals (McIsaac et al., 2016). Future studies could aim to obtain a higher sample number to mitigate, this issue (e.g., using a broader age range or identifying TBI through multiple datasets).

The findings from the current study have several important implications for the prevention and management of violence post-TBI. TBI should be recognised as a major risk factor for incarceration for violence. This highlights the need for routine TBI screening in health and criminal legal services (Admire & Mitchell, 2010; O’Brien, 2022; O’Rourke et al., 2017) to ensure individuals receive appropriate support to reduce the risk of violent offending. Furthermore, the increased risk associated with multiple treatment attempts may indicate a need for increased availability of TBI-specific interventions to ensure service users with TBI receive equitable treatment that addresses their needs whilst accounting for cognitive impacts and increased case complexity. Prior research suggests there is poor availability of evidence-based aggression and violence interventions for persons with ABI who are engaged in the criminal legal system (Guala et al., 2025). Interventions may also have reduced responsivity as individuals with TBI may encounter greater difficulty understanding the ramifications of offending behaviour and experience challenges achieving sustained behavioural change. Without evidence-based TBI-specific interventions to support correctional rehabilitation approaches, such efforts may have reduced benefit.

Notably, individuals with TBI who offend may struggle to access and remain engaged in health and social support following release from prison (Eriksson et al., 2019). This could explain the occurrence of multiple incarcerations and entrenchment of survivors of TBI within the criminal legal system. Violent offending could potentially be addressed by improving the provision of integrated support after prison contact. For example, Linkworker programs have shown preliminary effectiveness for improving post-release outcomes in samples of individuals with TBI (De Geus et al., 2021). Linkworkers support individuals with TBI exiting prison to access relevant health and social support services depending on their unique needs (De Geus et al., 2021; Ramos et al., 2018). Further evaluation of such services is warranted to determine their effectiveness over longer time periods.

This study identified that incarceration for violence is likely to occur within the first three years post-TBI, suggesting this time period is crucial to provide appropriate intervention that targets risk factors. Improved identification of health comorbidities in at-risk groups at the time-point of TBI and subsequent linkage to relevant support services could mitigate the risk of violent offending. Additionally, screening for a history of violence or legal involvement at the timepoint of TBI may provide valuable insight into potential risk of later incarceration for violence. This early risk assessment could inform targeted avenues for service provision where individuals can be directed to services designed to help with violence or other challenging behaviours linked to violence (e.g., positive behavioural support). That said, incarceration for violence occurred as early as three months post-TBI to just over 11 years post-TBI, highlighting that violence can be displayed in post-acute periods but can also emerge over a longer time-frame. Practitioners working within substance use and mental health services should be aware of the short and long-term risk of incarceration for violence after TBI and how risk may be further increased by comorbidities. This awareness could then inform more accurate risk assessment, case priority classification, and improved access to relevant treatment. For example, as per the risk need responsivity model (Andrews et al., 2006), individuals with higher levels of risk and greater case complexity are considered to be higher priority for intervention and should receive more intensive intervention.

There are several avenues for additional research to further expand on the current presented in the current study. Future research could build on this work by investigating whether there are specific substances that contribute to increased risk of incarceration for violence. The impact of substance types on violent offending may differ, for instance, (Håkansson & Jesionowska, 2018) found alcohol use to be associated with increased violent crime, whereas amphetamine use was not. Further exploration of specific substances used by individuals with TBI would be beneficial to identify key substances that increase the risk of violence in this population. Improved knowledge could inform the development of TBI-specific substance use interventions. Additionally, further investigation of the role of post-TBI mental health is warranted given the conflicting finding reported in the current study. As this study utilised administrative data, the presence of poor mental health may be underestimated. For example, individuals with TBI may not access support (e.g., due to service inaccessibility) but may still experience increased negative mental health outcomes post-TBI (Nagele et al., 2018; Silver & Nedelec, 2020). A potential direction for research may be investigating whether risk of incarceration for violence for young adults with TBI differs across individuals with poor mental health captured through formal support and individuals with mental health issues experienced in the absence of service contact.

The current study provides insight into the relationship between TBI and incarceration for violence by demonstrating that the increased risk is interconnected with health and social vulnerability. Ongoing offending, poor mental health, and substance use problems contribute to a considerably increased risk of incarceration for violence offences for young adults with TBI. To decrease incarceration for violence, TBI should be considered as a condition with chronic and often life-long impacts that require both short- and—long-term support for a range of health and social needs. Improved identification of TBI and appropriate support post-TBI could disrupt harmful cycles of ongoing criminal legal contact, reduce adverse health outcomes, and prevent incarceration for violence.

## Data Availability

The dataset analysed in the current study is not publicly available due to privacy regulations.

## Appendix

**Table 7:** ANZSOC classifications

Division	Subdivision	Descriptor
Division 01		Homicide and related offences
	0110	Murder
	0120	Attempted murder
	0131	Manslaughter
Division 02		Acts intended to cause injury
	0211	Serious assault resulting in injury
	0212	Serious assault not resulting in injury
	0213	Common assault
	0291	Stalking
	0299	Other acts intended to cause injury
Division 05		Abduction harassment and other offences against the person
	0511	Abduction and kidnapping
	0521	Deprivation of liberty/false imprisonment
	053	Harassment and threatening behaviour
	0531	Harassment and private nuisance
	0532	Threatening behaviour
Division 06		Robbery, extortion and related offences
	0611	Aggravated robbery

## Footnotes

1 Sexual offences/charges were excluded due to differences in this type of offending compared to non-sexual violent offending (Gudjonsson & Sigurdsson, 2000).

2 As the failure event requires entering prison for a violent offence, a person already in custody for another offence cannot experience it, therefore their hazard of incarceration for violence is lower during that period.
